# Semi-automated quantification of hard exudates in colour fundus photographs diagnosed with diabetic retinopathy

**DOI:** 10.1186/s12886-017-0563-7

**Published:** 2017-09-20

**Authors:** Abhilash Goud Marupally, Kiran Kumar Vupparaboina, Hari Kumar Peguda, Ashutosh Richhariya, Soumya Jana, Jay Chhablani

**Affiliations:** 1 Smt. Kanuri Santhamma Retina Vitreous Centre, L.V.Prasad Eye Institute, Hyderabad, 500034 India; 2 Engineering Group, Srujana Center for Innovation, L.V.Prasad Eye Institute, Hyderabad, 500034 India; 3 Deptartment of Electrical Engineering, Indian Institute of Technology Hyderabad, Hyderabad, 502285 India

**Keywords:** Hard exudates, Diabetic retinopathy, Colour fundus photography, Automated quantification, ImageJ, Macular edema, Disease management

## Abstract

**Background:**

Hard exudates (HEs) are the classical sign of diabetic retinopathy (DR) which is one of the leading causes of blindness, especially in developing countries. Accordingly, disease screening involves examining HEs qualitatively using fundus camera. However, for monitoring the treatment response, quantification of HEs becomes crucial and hence clinicians now seek to measure the area of HEs in the digital colour fundus (CF) photographs. Against this backdrop, we proposed an algorithm to quantify HEs using CF images and compare with previously reported technique using ImageJ.

**Methods:**

CF photographs of 30 eyes (20 patients) with diabetic macular edema were obtained. A robust semi-automated algorithm was developed to quantify area covered by HEs. In particular, the proposed algorithm, a two pronged methodology, involved performing top-hat filtering, second order statistical filtering, and thresholding of the colour fundus images. Subsequently, two masked observers performed HEs measurements using previously reported ImageJ-based protocol and compared with those obtained through proposed method. Intra and inter-observer grading was performed for determining percentage area of HEs identified by the individual algorithm.

**Results:**

Of the 30 subjects, 21 were males and 9 were females with a mean age of the 50.25 ± 7.80 years (range 33–66 years). The correlation between the two measurements of semi-automated and ImageJ were 0.99 and 0.99 respectively. Previously reported method detected only 0–30% of the HEs area in 9 images, 30–60% in 12 images and 60–90% in remaining images, and more than 90% in none. In contrast, proposed method, detected 60–90% of the HEs area in 13 images and 90–100% in remaining 17 images.

**Conclusion:**

Proposed method semi-automated algorithm achieved acceptable accuracy, qualitatively and quantitatively, on a heterogeneous dataset. Further, quantitative analysis performed based on intra- and inter-observer grading showed that proposed methodology detects HEs more accurately than previously reported ImageJ-based technique. In particular, we proposed algorithm detect faint HEs also as opposed to the earlier method.

## Background

**Fig. 1:**
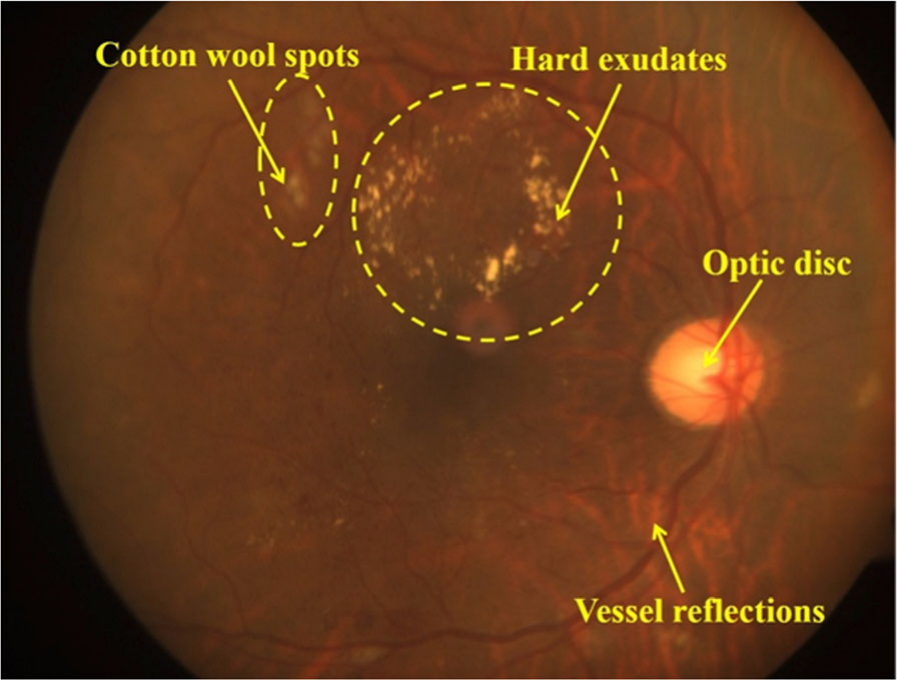
Sample colour fundus photograph diagnosed with diabetic retinopathy which depicts hard exudates alongside cotton wool spots. In this particular CF image, gray scale intensity distribution for HEs ranges from 85 to 200, for cotton wool spots from 80 to 130, for optic disc from 90 to 200 and for vessel reflections from 80 to 110

Diabetic retinopathy (DR) is the leading cause of blindness around the world and especially in developing countries [1, 2]. Early clinical signs of DR include hard exudates (HE), microaneurysms and retinal haemorrhages. In particular, HEs are the classical sign of DR; which are largely made of lipid residues of serous leakage from damaged capillaries and consists of lipid-laden macrophages or noncellular materials including lipid and proteinaceous substances [3–5]. In general, colour fundus photography (CF) is the one of the standard method to detect HEs (see Fig. 1). Studies show that CF provides a high sensitivity for a wide range of DR changes two dimensionally [6, 7]. So far, ophthalmologists assess the condition of the disease only qualitatively. However, qualitative evaluation may be useful for routine screening but may not be very useful while monitoring the treatment outcome. Specifically, clinicians seek quantitative analysis which enables them to perform accurate disease management. However, obtaining such information pose considerable challenge to clinicians. Difficulty arises because (i) the complex topology of HEs especially of the tiny ones makes it onerous for humans to demarcate manually and (ii) the number of HEs could be large in some images and hence manual scoring could be tedious, stressful and susceptible to human error. Against this backdrop, it is imperative to perform automated quantification of HEs based on CF images to facilitate accurate disease management.

Many attempts have been made towards automated detection of DR based on features obtained from HEs in CF images [8–25]. However, very limited efforts are made towards automated quantification of HEs. Sasaki et al. has reported a semi-automated method using ImageJ, a public domain image processing software [26]. Specifically, they employed maximum entropy thresholding on green colour plane of the CF images which results in detection of exudates along with few outliers. Subsequently, outliers were removed manually using selection box in ImageJ. However, applicability of this method on heterogeneous data is yet to be demonstrated. Further, performing all the steps including removing outliers sequentially by the observer is tedious. Against this backdrop, we propose a novel methodology for quantifying HEs in CF images of patients diagnosed with diabetic retinopathy and evaluated its accuracy on images with varying complexity. Further, we compared the proposed algorithm against the state-of-the art ImageJ-based method (Sasaki et al. [27]).

## Methods

This is a retrospective study conducted at the L V Prasad Eye Institute, Hyderabad, India. The study was approved by the institutional Review Board of the Institute and all the methods adhered to the tenets of the Declaration of Helsinki. The key inclusion criteria were subjects with presence of diabetic macular edema with presence of HEs. Subjects were excluded if they had coexisting ocular diseases which will affect the quality of colour fundus photographs (such as any media opacities). In this study we have included only good quality fundus images; a good quality image was defined as appropriately focused with good illumination which allowed clear identification of HEs. All subjects underwent comprehensive eye examination including best corrected visual acuity, slit lamp examination and dilated fundus examination by an ophthalmologist. Further, all the CF photographs were taken with 50^0^ protocol (FF 450 plus IR fundus camera; Carl Zeiss Meditec, Germany).

### Measurements of hard exudates by proposed algorithm

**Fig. 2:**
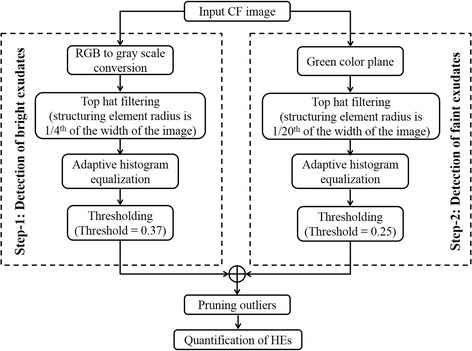
Schematic of proposed methodology

In general, HEs appear as yellow spots in the CF due to lipid deposits [3–5]. However, there exist other structures such as cotton wool spots, optic disc and retinal vessel reflections in CF whose intensity profile is quite similar to the HEs (refer to Fig. 1). In view of this, developing a fully automated algorithm to detect only HEs could be difficult. Therefore, we adopt a semi-automated methodology where outliers can be removed manually. Figure 2 depicts the schematic of the proposed methodology.

**Fig. 3:**
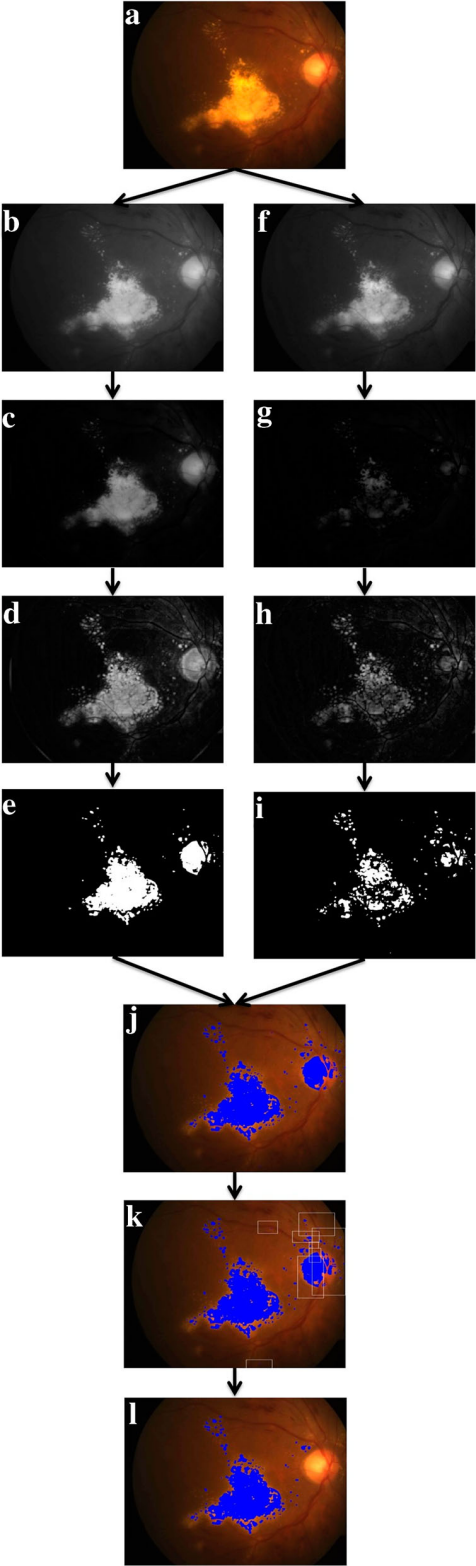
Graphical illustration of proposed algorithm: (**a**) Sample CF image; (**b**)--(**e**) images obtained after performing RGB to gray scale conversion, top-hat filtering, adaptive histogram equalization and thresholding in step-1, respectively; (**f**)--(**i**) images obtained after performing green plane extraction, top-hat filtering, adaptive histogram equalization and thresholding in step-2, respectively; (**j**) image after combing result of step-1 and step-2; (**k**) removing outliers using rectangular selection box and (**l**) image after removing outliers

In particular, a two pronged approach was adopted to detect both bright and weak/faint HEs. In step-1, to detect bright HEs, top-hat [26] filtering was first employed on the gray scale image (see Fig. 3b) of the CF using structuring element (disk, where radius of disk is considered to be one-fourth of the width of the image) to make background illumination more uniform (see Fig. 3c). Subsequently, adaptive histogram equalization [26] was performed to further enhance the exudates from background (see Fig. 3d). Then, the enhanced image was binarized using empirically determined threshold (0.37) to segment the exudates from background, in which the brighter pixels indicate the exudates (see Fig. 3e). However, in addition to exudates, some outliers were also resulted due to reflections from optic disc and retinal vessels in nerve fibre layer.

Next, in step-2, we proceed to detect weaker exudates that may not have been detected in step-1. Here, only green plane of the colour fundus image is considered because weak exudates are observed to be better contrasted in green plane than in gray scale image or red and blue planes (Fig. 3f). Then similar operations that were employed in step-1 were subsequently performed but with different thresholds. Specifically, radius of structuring element for performing top-hat filtering is decreased to one-twenty-fifth of the width of the image and threshold for binarization was empirically determined as 0.25 (Fig. 3g-i) for images obtained after respective operations). Step-2 also resulted in few outliers due the same reasons mentioned earlier in step-1.

Now, result of step-1 and step-2 is combined and outliers are removed manually using a rectangular selection box (Fig. 3j-k). Finally, the total area of the detected HEs was measured to facilitate monitoring the treatment response (Fig. 3l).

### Measurements of hard exudates by existing methodology using ImageJ

As alluded earlier, Sasaki et al. [27] previously reported a methodology for measurement of HEs using ImageJ software (version 1.51a; available in the public domain at https://imagej.nih.gov/ij/download.html Institute of Health, USA). In particular, CF image is split into red, green, blue channels (Image- > Colour- > Split Channels) and only green colour plane is selected. Subsequently, maximum entropy thresholding (Image- > Adjust- > Auto Threshold- > MaxEntropy) is applied on it which results in detection of exudates along with few outliers. Subsequently, outliers were removed manually using selection box in ImageJ. Accordingly, we analyzed the images using the same for comparison.

### Image grading

We now proceed to evaluating accuracy of the proposed methodology. In particular, for an algorithm to be deployed in clinical practice, it is important to quantify how much of the actual area covered by HEs is being detected by the algorithm. However, such quantification requires knowledge of actual HE area measurement or ground truth. Unfortunately, in this case, it is difficult to obtain ground truth measurements because it is generally obtained through manual demarcation of the HEs which may not be feasible for the reasons alluded earlier.

Against the above backdrop, we propose an alternative methodology, based on intuition, to quantify the ability of the proposed algorithm to identify HEs and to facilitate comparison between the proposed and ImageJ-based methods. To evaluate both the techniques of HEs measurement, we adopted an intuitive approach to quantify the ability of each algorithm to identify HEs (0–100%). In particular, intra and inter-observer grading was performed for determining percentage area of HEs identified by the individual algorithm. Two masked observers graded CF images twice after analyzed by both algorithms on separate sessions. Further, both the observers were masked to their own as well as other’s measurements. Two observations by each observer established the repeatability of the grading method, and comparison between two observers established the reproducibility of grading method. Finally, we consider the average of all four observers grading to facilitate comprehensive evaluation.

## Results

Thirty eyes from 20 subjects with diabetes were included the study. Twenty-one were males and nine were females with the mean age of 50.25 ± 7.80 years (range 33 to 66 years). Mean Best Corrected Visual Acuity (BCVA) was 0.454 ± 0.414 logMAR (Snellen equivalent 20/50). The mean duration of diabetes among study subjects was 10.96 ± 5.81 years. Distribution of diabetic retinopathy as per Early Treatment Diabetic Retinopathy Study (ETDRS) guidelines was, out of 30 non-proliferative diabetic retinopathy (NPDR) was present in 23 eyes and proliferative diabetic retinopathy (PDR) was present in 7 eyes. Among 23 eyes of NPDR, 6 eyes mild NPDR, 12 eyes moderate NPDR, and 5 eyes severe NPDR. The mean central macular thickness among study subjects was 418.26 ± 212.46 μm.

**Fig. 4:**
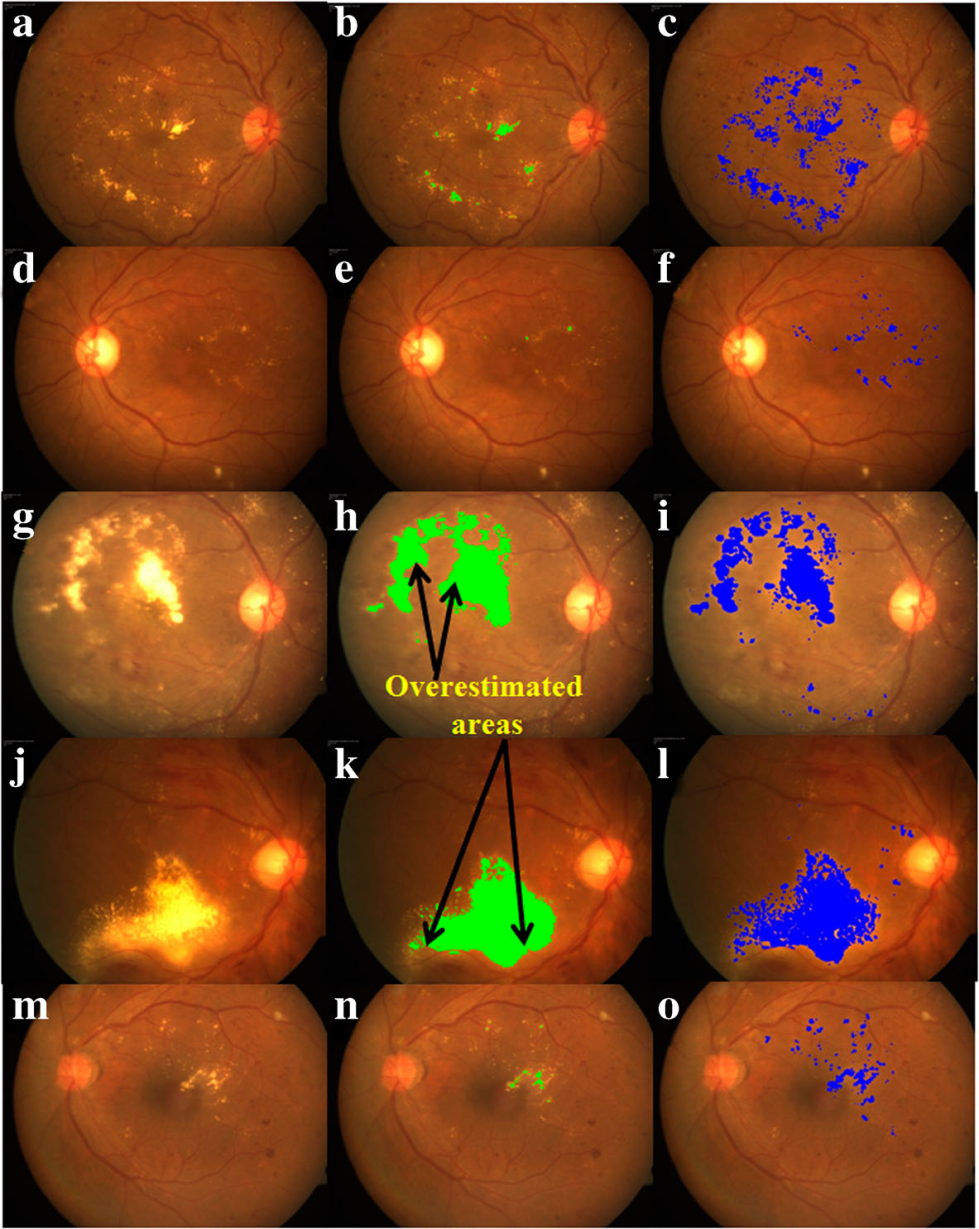
Qualitative comparison between ImageJ-based and proposed methods: Left-- Representative CF photographs of diabetic macular edema with HEs; Middle-- Detected HEs (indicated by green colour) by Sasaki’s ImageJ methodology; and Right-- Detected HEs (indicated by blue colour) by proposed algorithm

**Fig. 5:**
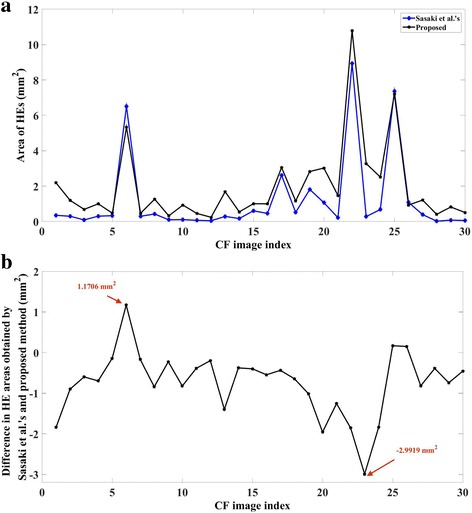
Area comparison: (**a**) HEs area obtained by ImageJ-based and proposed method, and (**b**) Corresponding difference

On five representative images, results obtained by proposed algorithm are furnished in Fig. 4 alongside segmentation obtained by ImageJ-based method for visual comparison which clearly depicts improved performance of the proposed algorithm. Further, proposed algorithm observed to detect more HEs vis-à-vis ImageJ-based. In particular, HEs area estimated by proposed algorithm varies from 0.2413 mm^2^ to 10.7865 mm^2^ with a mean of 1.9334 mm^2^ whereas HEs area obtained by ImageJ-based method varies from 0.0179 mm^2^ to 8.9327 mm^2^ with mean of 1.1853 mm^2^ (see Fig. 5a for further details). Further, difference in areas obtained by ImageJ-based method and proposed methods varies from −2.9919 mm^2^ to 1.1706 mm^2^ with a mean of −0.7481 mm^2^ (see Fig. 5b for further details). Except in one or two occasions, ImageJ-based method is found to underestimate the actual area of HEs as opposed to proposed algorithm. Further, it is observed that even in those cases where area obtained by ImageJ-based is greater than that of proposed method, the later is overestimating the area. In particular, for image indexed 6 in Fig. 5, extra areas detected by ImageJ-based method are depicted in Fig. 4h which contributes to the significant positive difference (1.1706 mm^2^) as seen in Fig. 5b.

### Statistical analysis

Next, we proceed to corroborate the proposed algorithm statistically. To this end, we begin with validating the observer grading evaluated on both the techniques, i.e., on proposed algorithm and ImageJ-based method. Subsequently, we compared the accuracy of HE area detection in both the algorithms.

### Validation of observer grading

**Fig. 6:**
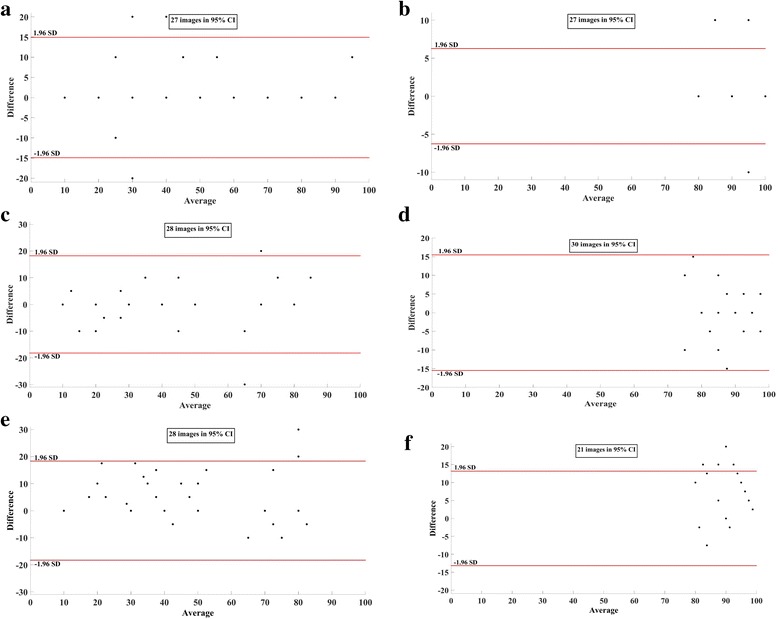
Bland-Altman plots: (**a**) Intra-observer repeatability of Grader A for images analyzed using ImageJ-based method, (**b**) Intra-observer repeatability of Grader A for images analyzed using proposed method, (**c**) Intra-observer repeatability of Grader B for images analyzed using ImageJ-based method, (**d**) Bland-Altman plot indicating repeatability of Grader B for images analyzed using proposed method, (**e**) Inter-observer repeatability between graders A and B for images analyzed using ImageJ-based method, and (**f**) Inter-observer repeatability between graders A and B for images analyzed using proposed method

To this end, we performed correlation coefficient (for two measurements *x*_*i*_ and *y*_*i*_, *i* = 1,2,...,*N*, correlation coefficient $(CC)={\sum}_{i=1}^N{x}_i\kern0.5em {y}_i/\sqrt{\sum_{i=1}^N{x}_i^2{\sum}_{i=1}^N{y}_i^2}\Big)$, and Bland-Altman analysis to evaluate repeatability of both intra- and inter-observer grading. Accordingly, for images analyzed on measurements obtained using ImageJ-based and proposed method, we obtained an ICC of 99.02 and 99.94%, respectively for grader-A, and an ICC of 98.27 and 99.60%, respectively for grader-B indicating intra-observer repeatability.. Further, inter-observer repeatability between average scoring of grader-A and grader-B for images analyzed on measurements obtained on ImageJ-based and proposed method 98.21 and 99.73%, respectively. In addition, Bland-Altman plots depicted in Fig. 6 also corroborates high intra-observer repeatability among two graders (Fig. 6a-d) and inter-observer repeatability for both the algorithms (Fig. 6e-f).

### Proposed algorithm vs ImageJ-based method

**Fig. 7:**
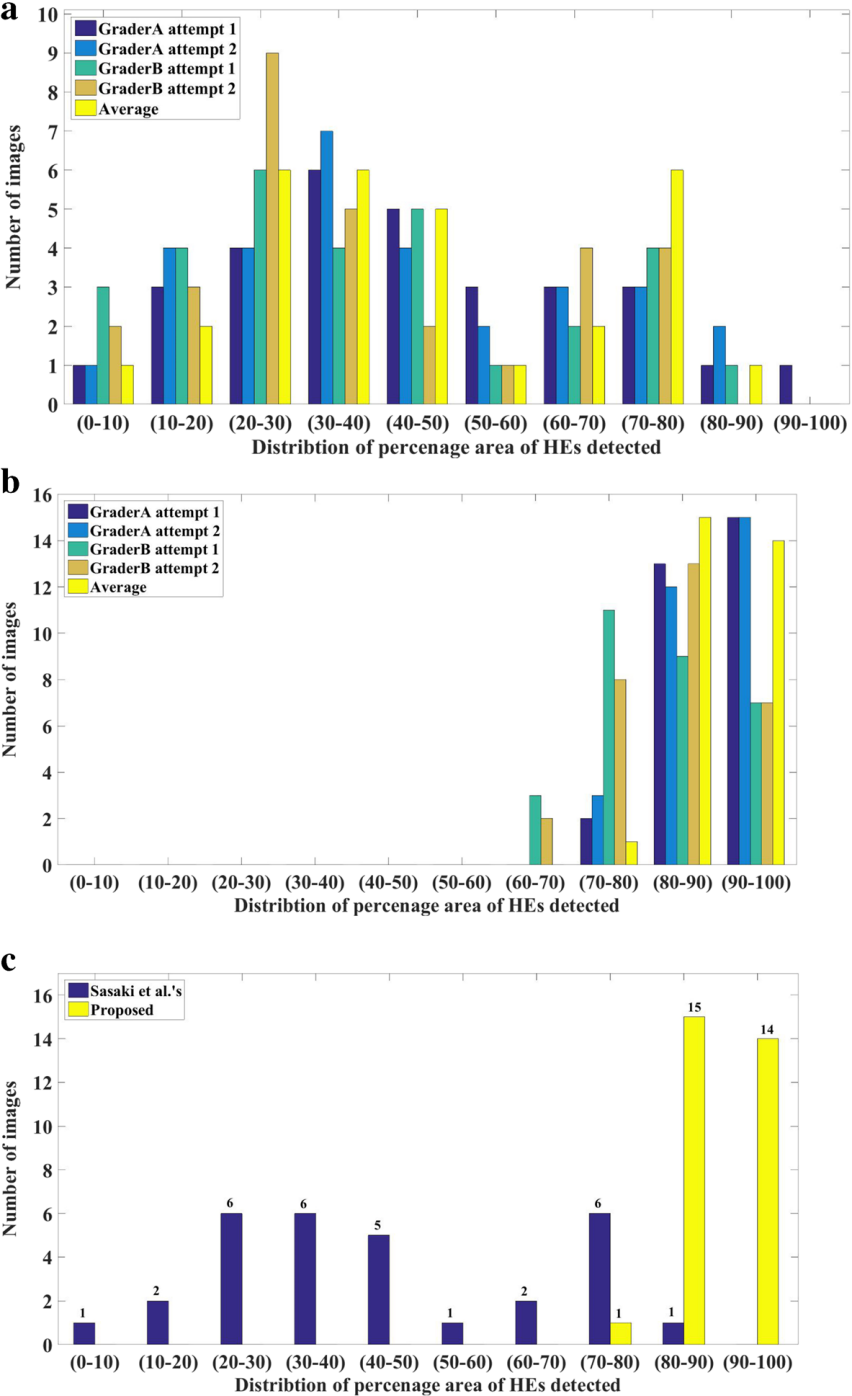
Statistical analysis: Distribution of percentage area of HEs obtained by (**a**) observer grading performed on images analysed using ImageJ-based ImageJ methodology, (**b**) observer grading performed on images analysed using proposed methodology, and (**c**) Average grading performed on images analysed using proposed methodology

In light of above observations which validates the observer grading, we now compare the performance of two algorithms. In particular, Fig. 7a presents distribution of HEs area graded by both the observers and twice by each observer for images analysed using ImageJ-based method. Further, average of all four grading is also included to facilitate unbiased evaluation. Notice that ImageJ-based algorithm can only detect 50% of the total HEs area or less. On the contrary, corresponding distributions for proposed algorithm, furnished in Fig. 7b, indicates that in most images proposed method is able to detect more than 80% of the HEs area. Fig. 7c summarizes the comparison of two techniques based on average grading. Specifically, ImageJ-based method detected only 0–30% of the HEs area in 9 images, 30–60% in 12 images and 60–90% in remaining images, but more than 90% in none. In contrast, proposed method, detected 60–90% of the HEs area in 13 images and 90–100% in remaining 17 images, buttressing the efficacy of the algorithm.

## Discussion

In this study, we proposed a semi-automated methodology to quantify the HEs. The algorithm is evaluated, qualitatively and quantitatively, on CF images taken from subjects diagnosed with DR. Further, algorithmic results are compared vis-à-vis state-of-the-art method based on ImageJ software. Qualitative evaluation shown that proposed technique detected HEs more accurately as opposed to ImageJ-based method. Subsequently, this observation is corroborated, quantitatively, with the help of statistical analysis. In particular, in the absence of ground truth quantification, we proposed an intuitive approach based on validated subjective grading, intra- and inter-observer, to evaluate both the techniques. Statistically, proposed algorithm detected more than 80% of the HEs in 96% of eyes whereas ImageJ-based method detected more than 80% HEs in only 3% of the eyes. More specifically, ImageJ-based method missed HEs, in 70% of eyes, up to 50%.

The ImageJ-based method performs sequence of steps including removing of spurious regions interactively which is slightly tedious, whereas the proposed method performs all operations automatically except removing outliers. ImageJ-based method uses max-entropy thresholding on the green channel of the colour fundus photograph which could detect only few bright hard exudates. In this regard, one of the main objectives of the proposed algorithm is to accommodate possible heterogeneity in the data which includes HEs with high variability in size as well as contrast. To this end, the proposed algorithm uses two pronged methodology to detect both bright and faint hard exudates. In particular, specific pre-processing steps which include top-hat filtering and adaptive histogram equalization are performed to make hard exudates regions more distinguishable from background. Further, for thresholding, distinct empirically determined thresholds are employed for bright and faint exudates. Accordingly, the dataset considered includes CF images with varying contrast levels. Further, HEs present are of different sizes ranging from as small as 0.2413 mm^2^ to as big as 10.7865 mm^2^. Desirably, experimental results showed that proposed algorithm performed well in almost all the images, whereas ImageJ-based method does not accommodate the variations and as a result failed to detect a large numbers HEs especially the faint ones.

Although the current algorithm is designed to estimate overall area of the HEs, it is capable of estimating individual HE areas with minor software modifications. In the recent past, various clinical studies evaluated HE resolution with lipid lowering agents as well as with intravitreal therapy [28–30], however, evaluation was done using ETDRS grids, which is not accurate and precise. Our algorithm provides accurate and reproducible method of quantifying HEs. This can be used for treatment monitoring as well as management decisions.

One of the limitation of current semi-automated technique is that it may appear slightly tedious due to manual corrections but it facilitates to obtain accurate quantification by removing spurious areas. In near future, we plan to make the algorithm fully automated by employing machine learning approaches to classify HEs from cotton wool spots, optic disc and other reflections.

The proposed algorithm can also be used to detect drusens in CF images. However, performance evaluation is yet to be completed. To this end, we envisage to perform thorough statistical analysis to explore possibility of application of the algorithm in clinical studies based on drusen quantification.

## Conclusion

In summary, proposed semi-automated algorithm detects HEs more accurately than previously reported technique based on ImageJ software. Further, it was observed the proposed algorithm is able to detect even faintly visible exudates which are not detected by previous method. Future applications of this algorithm in quantification of HEs in diagnosis and monitoring of eyes with diabetic macular edema may contribute further in deciding management strategies.

## Abbreviations

(BCVA): Best corrected visual acuity
(CF): Color fundus photography
(DR): Diabetic Retinopathy
(DR): Proliferative Diabetic Retinopathy
(ETDRS): Early treatment Diabetic Retinopathy Study
(HEs): Hard exudates
(NPDR): Non proliferative diabetic retinopathy
